# Maternal Prenatal Inflammation Increases Brain Damage Susceptibility of Lipopolysaccharide in Adult Rat Offspring via COX-2/PGD-2/DPs Pathway Activation

**DOI:** 10.3390/ijms23116142

**Published:** 2022-05-30

**Authors:** Jiahua Zhang, Peishuang Yao, Wenli Han, Ying Luo, Yuke Li, Yang Yang, Hui Xia, Zhihao Chen, Qi Chen, Hong Wang, Lu Yang, Huan Li, Congli Hu, Haifeng Huang, Zhe Peng, Xiaodan Tan, Miaomiao Li, Junqing Yang

**Affiliations:** 1 Department of Pharmacology, The Key Laboratory of Biochemistry and Molecular Pharmacology, Chongqing Medical University, Chongqing 400016, China; aimee08221205@163.com (J.Z.); 2020111589@stu.cqmu.edu (P.Y.); 100894@cqmu.edu.cn (Y.L.); liyuke@126.com (Y.L.); cqfyyy2020@163.com (Y.Y.); xiah0207@126.com (H.X.); miachen6169@gmail.com (Z.C.); chenqi@gz5055.com (Q.C.); 101832@cqmu.edu.cn (H.W.); Aluyoung@163.com (L.Y.); 13996433553@163.com (H.L.); hucongli1217@126.com (C.H.); 2017010178@stu.cqmu.edu.cn (H.H.); pengzsunshine@163.com (Z.P.); govancha@163.com (X.T.); meetmiaomiao@163.com (M.L.); 2 Animal Laboratory Center, Chongqing Medical University, Chongqing 400016, China; 0904hanwenli@163.com

**Keywords:** prenatal maternal inflammation, lipopolysaccharide, central nervous system inflammation, cyclooxygenase-2, DPs

## Abstract

A growing body of research suggests that inflammatory insult contributes to the etiology of central nervous system diseases, such as depression, Alzheimer’s disease, and so forth. However, the effect of prenatal systemic inflammation exposure on offspring brain development and cerebral susceptibility to inflammatory insult remains unknown. In this study, we utilized the prenatal inflammatory insult model in vivo and the neuronal damage model in vitro. The results obtained show that prenatal maternal inflammation exacerbates LPS-induced memory impairment, neuronal necrosis, brain inflammatory response, and significantly increases protein expressions of COX-2, DP2, APP, and Aβ, while obviously decreasing that of DP1 and the exploratory behaviors of offspring rats. Meloxicam significantly inhibited memory impairment, neuronal necrosis, oxidative stress, and inflammatory response, and down-regulated the expressions of APP, Aβ, COX-2, and DP2, whereas significantly increased exploring behaviors and the expression of DP1 in vivo. Collectively, these findings suggested that maternal inflammation could cause offspring suffering from inflammatory and behavioral disorders and increase the susceptibility of offspring to cerebral pathological factors, accompanied by COX-2/PGD-2/DPs pathway activation, which could be ameliorated significantly by COX-2 inhibitor meloxicam treatment.

## 1. Introduction

Alzheimer’s disease (AD) is an age-dependent progressive neurodegenerative disorder marked by overall deficits in cognition function [1]. Several hypotheses have been reported to explain the complex pathophysiological conditions of AD such as amyloid cascade, oxidative stress, excitotoxicity, and neuroinflammation [2]. Compelling evidence suggests that neuroinflammation is an important part of Alzheimer’s disease (AD) pathogenesis, associated with obviously increased amyloid-β (Aβ) aggregates [2,3]. During the inflammatory reaction-associated AD, the ramified/inactive microglia cells become activated and secrete high levels of proinflammatory cytokines [3]. Inflammatory cytokines such as interleukin (IL)-1β, IL-6, tumor necrosis factor-α (TNF-α), or transforming growth factor-β (TGF-β) can augment amyloid precursor protein (APP) expression and Aβ formation [4]. Furthermore, clinical studies have also shown a definite association between infection and increased morbidity of dementia in the elderly, and infection could triple the morbidity of AD over 5 years [5]. Inflammatory molecules including C-reactive protein and inerleukin-6 are elevated in blood years before dementia [6,7]. The gram-negative bacterial cell wall-derived LPS is a potent inflammatory agent; it can promote the formation of amyloid-like plaques in rat brain [8,9]. In a previous paper, chronic LPS infusion via lateral ventricle was suggested to constitute a useful animal model to study the inflammatory reaction associated with the pathology of Alzheimer’s disease [10]. However, the underlying mechanisms involved in LPS’s accelerated formation of amyloid-like plaques and induced cognitive impairment are not known.

In humans, epidemiologic research has indicated that early life challenges, as result of perinatal exposure to infection, can have long-lasting, negative effects on neurochemistry, brain excitability, and behavior [11]. Likewise, the recent genome-wide association study has identified an obvious correlation between the activation of the maternal immune system and the incidence of AD-like brain damage in the offspring [7,12]. Although perinatal care has greatly improved the survival of newborns from infected mothers, maternal inflammation could still arrest neural development and impair brain functions of the offspring [13]. In the rhesus monkey model, maternal infection at the end of the first trimester results in social behavioral abnormalities [14]. As previously reported, fetal mice’s exposure to maternal infection led to other behavioral abnormalities, including enhanced repetitive behaviors (increased marble burying) and increased anxiety (decreased time spent in the center of an open field arena) in adult male offspring [15]. Taken together, these studies show that maternal infection contributes to behavioral abnormalities associated with a higher risk of autism and schizophrenia in both primate and rodent offspring. Accordingly, we hypothesize that maternal inflammation may increase the risk of AD-like cognitive dysfunction in the offspring of adult rats. To elucidate the early role of inflammatory processes in the development of AD-like pathology in rats, we used LPS to stimulate the immune system of our experimental animals and sought to determine its underlying mechanisms.

Exposure to LPS during pregnancy is known to alter the activity of the hypothalamic–pituitary–adrenocortical (HPA) axis and cause a reduction in mesencephalic dopaminergic neurons in the brain of the offspring [16]. Moreover, a study demonstrated that LPS exposure during a critical time of embryonic development could produce long-term reduction in DA and 5-HT and other neurophysiological changes [17]. Interestingly, for the maternal injection of LPS at embryonic gestation day 11 (E11), E12 and E13 cause fetal death in a dose-dependent manner via an increase in cyclooxygenase-2 (COX-2) expression in the decidua [18]. These studies have focused on developing HPA, DA, and 5-HT; however, surprisingly, research on maternal inflammation-induced COX-2 changes in the brain of offspring is limited. Hence, we investigated for the first time the effects of a rat model of prenatal maternal infection during pregnancy on cognitive dysfunction in offspring to determine whether these effects are dependent on the change of COX2 in the brain of offspring.

Cyclooxygenase (COX), a rate-limiting enzyme in the synthesis of PGs, regulates multiple functions of the central nervous system (CNS) [19]. COX generally includes the structural COX-1 and the inducible COX-2, and the latter, which can be induced by LPS or cytokines, is found to work in concert in a number of similar pathophysiological activities and inflammatory disease [20]. A wide spectrum of studies has indicated that levels and enzymatic activity of COX-2 are markedly induced and lead to neuronal damage in stroke, neurodegenerative diseases such as Parkinson’s disease, and amyotrophic lateral sclerosis [21]. Our previous studies also proved that overexpressed COX-2 was involved in chronic brain damage in aluminum-overloaded rats [22]. In addition, studies have shown that COX-2 inhibitors (meloxicam, MXC) can alleviate neuronal damage, suggesting that AA epoxidase pathway metabolites may also play a key role in neuronal damage during brain inflammation [23]. These above-described results implied that increased enzymatic activity of COX-2 represents a common mechanism of neurotoxicity that underlies a wide range of acute and chronic neurological diseases. However, the effect of COX-2 and its downstream pathway on brain damage in rat offspring of prenatal exposure to LPS remains largely unclear. Thus, the understanding of mechanisms involved in the increase in COX-2 leading to brain damage may be exploited for the development of strategies for neuroprotection against the effects of such insults.

COX-2 exerts its physiological effects by producing PGs, such as prostaglandin D2 (PGD2), prostaglandin E2 (PGE2), etc. In peripheral tissues, PGD2 executes a wide range of functions, including vasodilatation, inhibition of platelet aggregation, allergic reaction mediation, and intraocular pressure reduction [24,25]. PGD2 is the most abundant type of PG in the brain and has been shown to contribute to sleep induction, the modulation of body temperature, olfactory function, hormone release, nociception, and neuromodulation [26]. PGD2 has been reported to provide neuroprotective effects during acute brain damage [27]. Additionally, the PGD2 of physiological concentration has been shown to rescue neurons from excitatory toxicity in vitro [28]. However, PGD2 has also been demonstrated to significantly cause hippocampus neuronal apoptosis [29]. PGD2 exerts its physiological effects by directly activating the heterotrimeric G-protein-coupled receptors, prostaglandin D1 (DP1) and prostaglandin D2 (DP2) [30]. Epidemiology shows that DP1 and DP2 expressions are varied in different organs and diseases. In recent years, it has been documented that the DP1 agonist can significantly reduce neuronal death caused by N-methyl-D-aspartic acid (NMDA) treatment in primary neurons and hippocampus slices [27]. In contrast, DP2 has high expression and excitotoxicity in normal hippocampus pyramidal neurons, and DP2 agonists can significantly enhance the damage of CA1 neurons induced by glutamate toxicity [31]. According to the above-described findings, the role of DP1 and DP2 in neuronal damage is controversial. Furthermore, the effects of the COX-2/PGD2/DPs’ pathway intervention on the susceptibility of brain damage in progeny rats with maternal inflammation have not been examined directly.

Therefore, we hypothesize that maternal inflammation could trigger inflammatory responses in offspring’s brains, and hence increase the susceptibility of offspring neurons to pathological factors, which is accompanied by activation of the COX-2/PGD-2/DPs pathway. The validation of the hypothesis would give rise to a further understanding of the susceptibility mechanism of brain damage caused by inflammation in pregnancy. In addition, it would provide an important experimental and theoretical basis to find new targets for brain damage, and to develop effective drugs for the prevention and treatment of CNS damage.

## 2. Results

### 2.1. Effect of MXC on Learning Cognitive Function in LPS-Treated Offspring from Prenatal Inflammation Exposure

**Figure 1:**
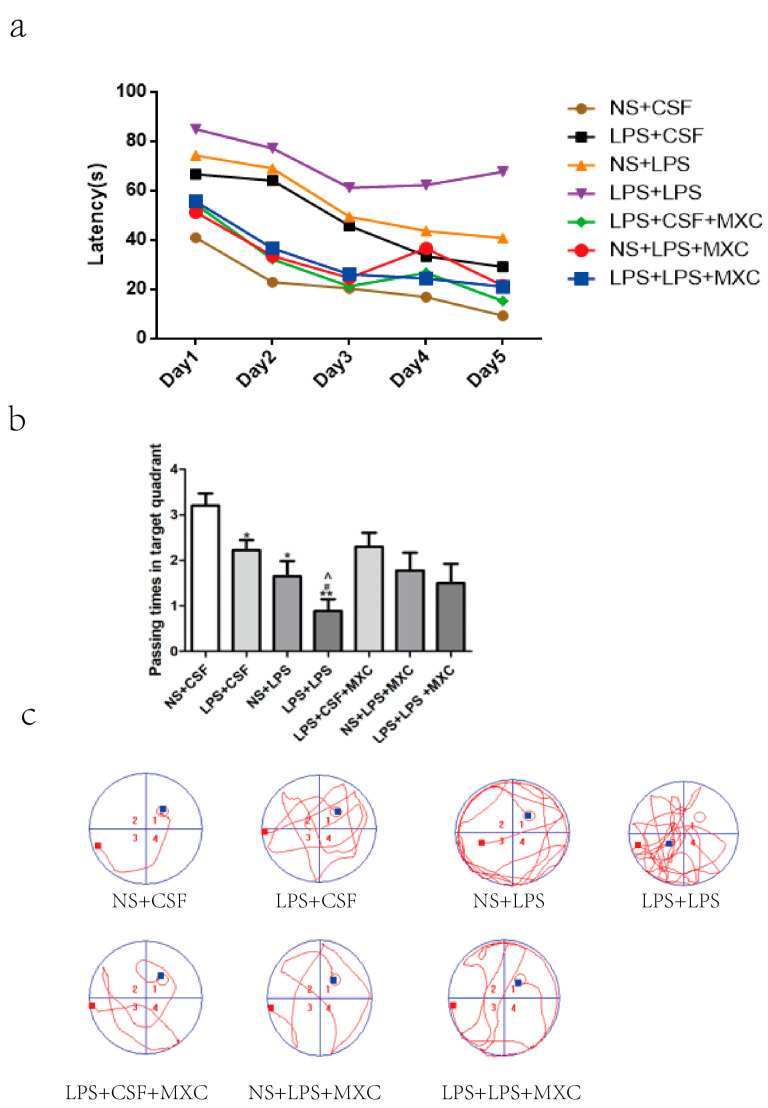
Effect of MXC on learning cognitive function in LPS-treated offspring from prenatal inflammation exposure: (**a**) Comparison of the mean escape latencies to find the hidden platform at days 1–5. Escape latencies were significantly increased in LPS + CSF, NS + LPS, and LPS + LPS groups compared with NS + CSF group. Compared with NS + LPS group, LPS + LPS group animals spent more time in finding. (**b**) Comparison of the mean in the number of crosses in the MWM at day 5. Passing times in target quadrant were significantly decreased in LPS + CSF, NS + LPS, and LPS + LPS groups compared with NS + CSF group. Data are mean ± SEM, *n* = 9–10. (* *p* < 0.05, ** *p* < 0.01 vs. NS + CSF group; # *p* < 0.05 vs. LPS + CSF group; ^ *p* < 0.05). (**c**) Comparison of each group to find the trajectory of the hidden platform.

As showed in (Figure 1), the behavior of male rats at PD60 changed significantly, and there was a significant difference in the escape latency of different groups. The results showed that escape latency significantly increased in LPS + CSF, NS + LPS, and LPS + LPS groups (*p* < 0.05 and *p* < 0.01) compared with the NS + CSF group. Rats from the “two-hit” LPS + LPS group spent more time in finding the platform than those from postnatal inflammation (NS + LPS) (*p* < 0.05) and prenatal inflammation (LPS + CSF) (*p* < 0.05) groups, and no significant differences were found between the NS + LPS group and the LPS + CSF group. There was a significant difference in the escape latency between the LPS + CSF group and the LPS + CSF + MXC group (*p* < 0.05 and *p* < 0.01). Compared with the NS + LPS group, the escape latency was clearly decreased in the NS + LPS + MXC group (*p* < 0.05 and *p* < 0.01). Compared with the LPS + LPS group, the escape latency was decreased significantly in the LPS + LPS + MXC group (*p* < 0.05 and *p* < 0.01) (Figure 1a).

Passing times in the target quadrant were significantly decreased in LPS + CSF, NS + LPS, and LPS + LPS groups (*p* < 0.05 and *p* < 0.01) compared with the NS + CSF group. Passing times of the LPS + LPS group were obviously decreased than those from postnatal inflammation (NS + LPS) (*p* < 0.05) and prenatal inflammation (LPS + CSF) (*p* < 0.05) groups. Passing times in the target quadrant tend to decrease in the NS + LPS group compared with the LPS + CSF group, and no obvious differences were observed between the LPS + CSF group and the LPS + CSF + MXC group. Passing times in the target quadrant tend to increase in the NS + LPS + MXC group compared with the NS + LPS group. Compared with the LPS + LPS group, passing times in the target quadrant were increased significantly in the LPS + LPS + MXC group (*p* < 0.05 and *p* < 0.01) (Figure 1b).

The analysis of male rats’ performance at PD60 showed a significant main effect of prenatal treatment. The LPS + CSF group achieved less path efficiency and travelled a greater distance to the platform and spent more time in the peripheral ring compared with NS + CSF group. Compared to NS + LPS group, the LPS + LPS group prolonged latency to the platform, and the first path length to the target zone was also increased. Meloxicam-treated rats reduced the escape distance compared to the LPS + LPS group throughout all test days (Figure 1c).

### 2.2. Effect of MXC on the Exploratory Behavior and Spontaneous Locomotor Activity in LPS-Treated Offspring from Prenatal Inflammation Exposure

**Figure 2:**
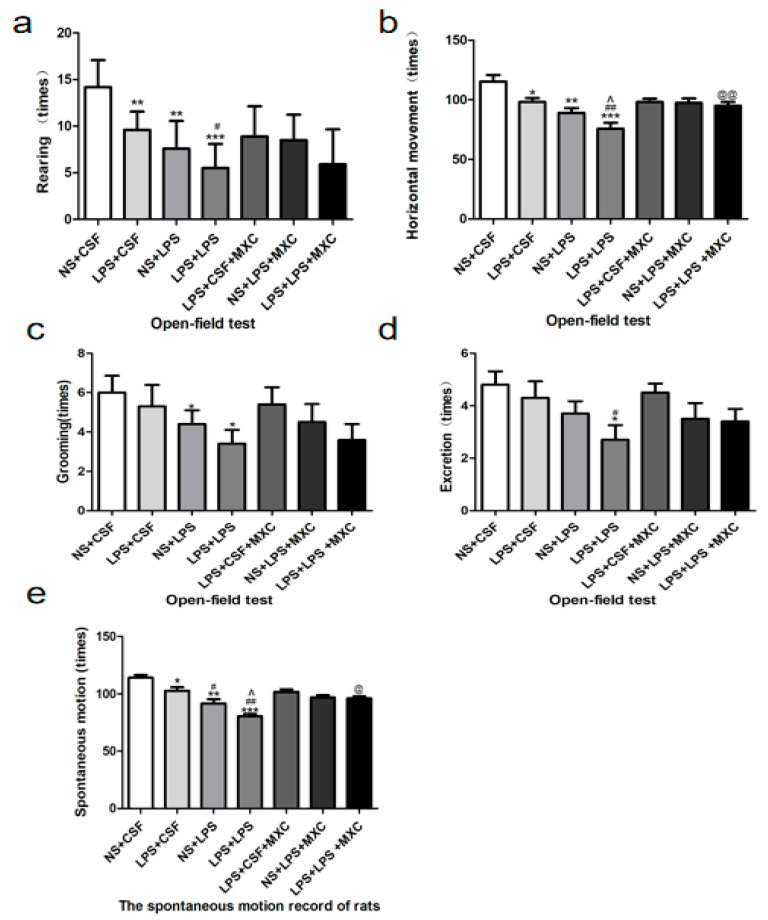
Effect of MXC on the exploratory behavior and spontaneous locomotor activity in LPS-treated offspring after prenatal inflammation exposure: (**a**) rearing behavior, (**b**) horizontal movement (times), (**c**) grooming episodes, and (**d**) amount of defecation during the 5 min trial period. (**e**) Effect of MXC on the spontaneous motor activity in LPS-treated offspring from prenatal inflammation exposure. Data are mean ± SEM, *n* = 9–10. (* *p* < 0.05, ** *p* < 0.01, *** *p* < 0.001 vs. NS + CSF group; # *p* < 0.05, ## *p* < 0.01 vs. LPS + CSF group; ^ *p* < 0.05 vs. NS + LPS group; @ *p* < 0.05, @@ *p* < 0.01 vs. LPS + LPS group).

Rats treated with LPS demonstrated horizontal movement, rearing, engaged with the grooming, and defecation were clearly lower, as compared to the NS + CSF group (Figure 2). Prenatal inflammation (LPS + CSF) demonstrated that rearing (*p* < 0.05) (Figure 2a) and horizontal movement (*p* < 0.05) (Figure 2b) were clearly decreased, as compared to the NS + CSF group; no significant effects were achieved for the grooming and defecating behaviors. Compared with LPS + CSF group, the rearing (*p* < 0.05) (Figure 2a), horizontal movement (*p* < 0.05) (Figure 2b), and defecating behaviors (*p* < 0.05) (Figure 2d) were clearly reduced in the LPS + LPS group; no significant effects were achieved for the grooming. Additionally, compared with the NS + LPS group, horizontal movement was markedly reduced in the LPS + LPS group (*p* < 0.05) (Figure 2b); however, no significant effects were achieved for the grooming, the rearing, and defecating behaviors. Rats treated with meloxicam demonstrated horizontal movement to be higher as compared to the LPS + LPS group. These results confirm that LPS decreases the locomotor activity of rodents in the open-field test. Previous studies have also shown that meloxicam can also increase locomotor activity.

Spontaneous locomotor (Figure 2d) was markedly decreased in LPS + CSF, NS + LPS, and LPS + LPS groups (*p* < 0.05 and *p* < 0.01) compared with the NS + CSF group. Significantly reduced spontaneous locomotor activity was observed in the NS + LPS and LPS + LPS groups (*p* < 0.05 and *p* < 0.01) compared with the LPS + CSF group. Significantly decreased spontaneous locomotor activity was observed in the LPS + LPS group (*p* < 0.05) compared with the NS + LPS group. Meloxicam-treated rats increased the spontaneous locomotor activity compared to the LPS + LPS group throughout all test days. Nevertheless, the results of the YLS-1B test indicate that meloxicam has an enhancing effect on general locomotor activity and does not induce any locomotor impairment (Figure 2e).

### 2.3. Effect of MXC on Hippocampal and Cortical Histopathology in LPS-Treated Offspring from Prenatal Inflammation Exposure

**Figure 3:**
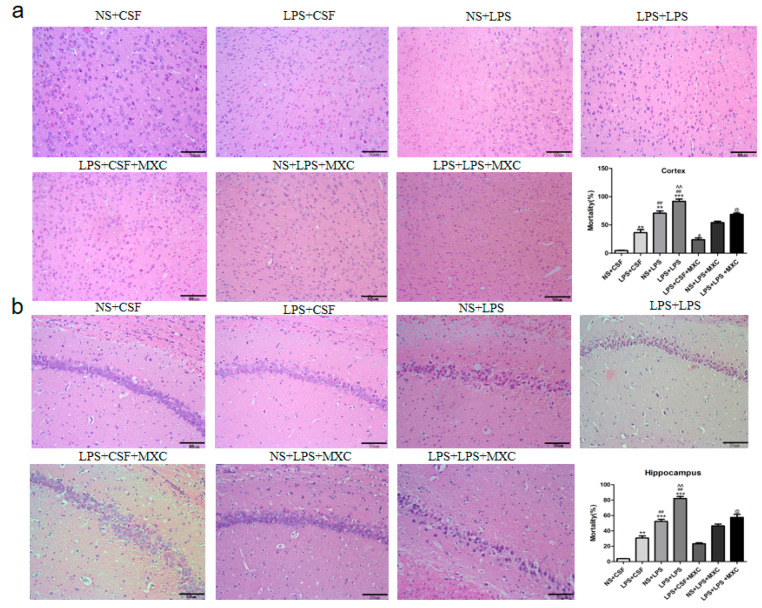
Effect of MXC on hippocampal and cortical histopathology in LPS-treated offspring from prenatal inflammation exposure: (**a**) Representative images of HE staining in cortex fields from NS + CSF, LPS + CSF, NS + CSF, LPS + CSF, LPS + CSF + MXC, NS + CSF + MXC, LPS + CSF + MXC groups; quantitative analysis of neuronal counts in the seven groups. Note decreased neuronal counts and enhanced neuronal damage in LPS + LPS group compared with NS + LPS group. (**b**) Representative images of HE staining in hippocampus fields from NS + CSF, LPS + CSF, NS + CSF, LPS + CSF, LPS + CSF + MXC, NS + CSF + MXC, LPS + CSF + MXC groups; quantitative analysis of neuronal counts in the seven groups. Note reduced neuronal counts and increased neuronal damage in LPS + LPS group compared with NS + LPS group. Data are mean ± SEM, *n* = 4. (** *p* < 0.01, *** *p* < 0.001 vs. NS + CSF group; # *p* < 0.05, ## *p* < 0.01 vs. LPS + CSF group; ^^ *p* < 0.01 vs. NS + LPS group; @ *p* < 0.05 vs. LPS + LPS group).

In the central nervous system, the hippocampus and cortex are important brain regions closely related to learning, memory, and behavioral and emotional functions. In HE-stained sections, the cortex woven nerve cells of the NS + CSF group were clear, well-structured, and neatly arranged, and no obvious nuclear pyknosis was detected. In all model groups, cell loss and the nuclear pyknosis of cortex neurons in rats were more than those in group NS + CSF. The results revealed the significant effects of both prenatal and postnatal treatments on neuronal counts in the cortex field. Furthermore, there was a sizable prenatal–postnatal treatment interaction. Compared with the NS + CSF group, analysis showed that neuronal counts obviously increased in LPS + CSF, NS + LPS, LPS + LPS groups (*p* < 0.01). Significant differences in the effects of the neuronal counts in the cortex field were observed between prenatal (LPS + CSF) and postnatal (NS + LPS) treatments. The postnatal LPS challenge caused greater neuronal loss in the cortex in the NS + LPS group (*p* < 0.05) compared with the LPS + CSF group. LPS + LPS rats (*p* < 0.05) demonstrated increased neuronal damage compared with NS + LPS rats. Administration of the meloxicam group significantly increased neuronal counts compared to the LPS + LPS group (Figure 3a). Results for neuronal counts in the hippocampus field were similar to those of the cortex field, as showed in Figure 3b.

### 2.4. Effect of MXC on the Levels of PGD-2, TNF-α, IL-1β and IL-6 in LPS-Treated Offspring from Prenatal Inflammation Exposure

**Figure 4:**
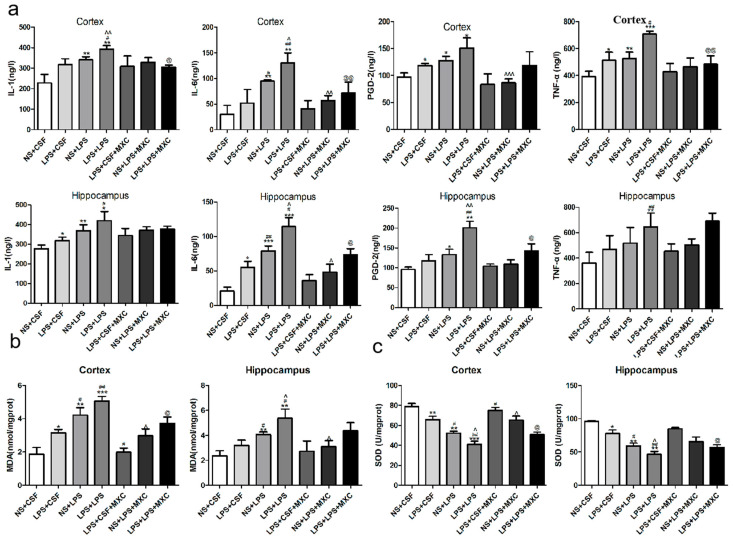
Effect of MXC on the levels of inflammatory factors and oxidative stress in LPS-treated offspring from prenatal inflammation exposure: (**a**) Effect of MXC on the levels of PGD-2, TNF-α, IL-1β, and IL-6 in LPS-treated offspring from prenatal inflammation exposure. Different protein expressions levels were determined through ELISA kits. (**b**) Effect of MXC on the MDA levels in LPS-treated offspring after prenatal inflammation exposure. (**c**) Effect of MXC on the SOD activity in LPS-treated offspring after prenatal inflammation exposure (right). Data are mean ± SEM, *n* = 4. (* *p* < 0.05, ** *p* < 0.01, *** *p* < 0.001 vs. NS + CSF group; # *p* < 0.05, ## *p* < 0.01 vs. LPS + CSF group; ^ *p* < 0.05, ^^ *p* < 0.01, ^^^ *p* < 0.001 vs. NS + LPS group; @ *p* < 0.05, @@ *p* < 0.01 vs. LPS + LPS group)**.**

To explore whether PGD-2, TNF-α, IL-1β, and IL-6 could be involved in LPS-induced neuroinflammation, we tested PGD-2, TNF-α, IL-1β, and IL-6 contents in the cortex and hippocampus after LPS treatment. Compared with NS + CSF group, the levels of PGD-2 and TNF-α in cortex were obviously increased in the LPS + CSF group (*p* < 0.05); however, there was no significant difference in the contents of IL-1β and IL-6 in the cortex. In the NS + LPS group and in the LPS + LPS group, the levels of TNF-α, IL-1β, and IL-6 in the cortex were significantly increased compared with the NS + CSF group and the LPS + CSF group, respectively (*p* < 0.05 and *p* < 0.01). Additionally, compared with the NS + LPS group, the levels of IL-1β and IL-6 in the cortex were significantly increased in the LPS + LPS group (*p* < 0.01); however, there was no significant difference in the contents of PGD-2 and TNF-α in cortex. Compared to the LPS + LPS group, the levels of TNF-α, IL-1β, and IL-6 were reduced markedly in LPS + LPS + MXC (*p* < 0.05 and *p* < 0.01) (Figure 4a). Results for these inflammatory factors in the hippocampus were similar to those of cortex showed in (Figure 4b).

### 2.5. Effect of MXC on the MDA Levels and the SOD Activity in LPS-Treated Offspring from Prenatal Inflammation Exposure

Compared with NS + CSF group, the levels of MDA in the cortex were significantly increased in LPS + CSF, NS + LPS, and LPS + LPS groups (*p* < 0.05 and *p* < 0.01), and no significant effects were achieved for the levels of MDA in the hippocampus between the NS + CSF group and the LPS + CSF group. In the NS + LPS group and the LPS + LPS group, the levels of MDA in the cortex and hippocampus were clearly increased compared with the LPS + CSF group (*p* < 0.05 and *p* < 0.01). Furthermore, compared with the NS + LPS group, the levels of MDA in cortex and hippocampus were significantly increased in the LPS + LPS group (*p* < 0.05). Compared with LPS + LPS group, the content of MDA in cortex of meloxicam-treated rats clearly decreased (*p* < 0.05) (Figure 4b). However, compared with NS + CSF group, the activity of SOD in the cortex and hippocampus were significantly decreased in LPS + CSF, NS + LPS, and LPS + LPS groups (*p* < 0.01). In the NS + LPS group and the LPS + LPS group, the SOD activity in the rat cortex and hippocampus was significantly decreased compared with the LPS + CSF group (*p* < 0.05). In addition, compared with the NS + LPS group, the activity of SOD in the cortex and hippocampus was obviously decreased in the LPS + LPS group (*p* < 0.05). The activity of SOD was significantly increased in meloxicam-treated rats compared to the LPS + LPS group in the cortex and hippocampus (*p* < 0.05) (Figure 4c).

### 2.6. Effect of MXC on the Protein Expressions of COX-2, DP2, DP1, Aβ, and APP in LPS-Treated Offspring from Prenatal Inflammation Exposure

**Figure 5:**
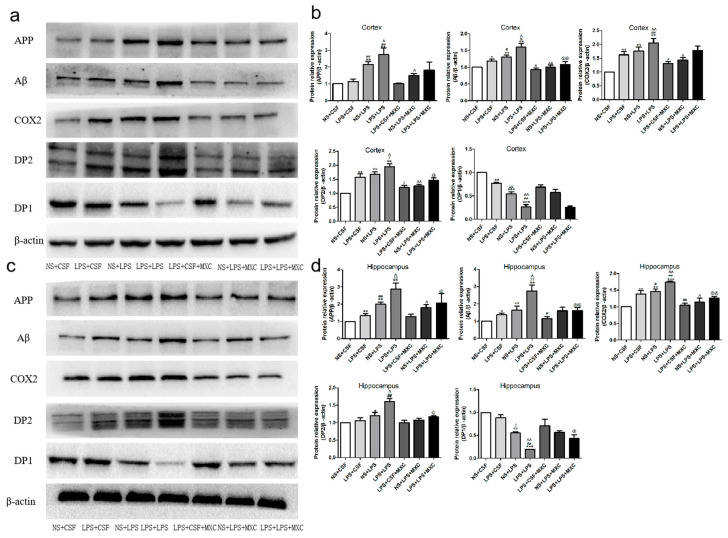
Western blot analysis of the relative protein contents in the cortex and hippocampus: (**a**) Western blot analysis of the relative protein contents in the cortex. (**b**) The expressions of APP, Aβ, COX-2, DP2, and DP1 were detected by Western blotting using specific antibodies in the cortex of rats. Protein contents were plotted for NS + CSF, LPS + CSF, NS + LPS, LPS + LPS, LPS + CSF + MXC, NS + LPS + MXC, and LPS + LPS + MXC. For APP, Aβ, COX-2, DP2, and DP1 proteins, the relative optical density was normalized to β-actin. Each result of the NS group is normalized to 1. (**c**) Western blot analysis of the relative protein contents in the hippocampus. (**d**) The expressions of APP, Aβ, COX-2, DP2, and DP1 were detected by Western blotting using specific antibodies in the hippocampus of rats. Protein contents were plotted for the NS + CSF, LPS + CSF, NS + LPS, LPS + LPS, LPS + CSF + MXC, NS + LPS + MXC, and LPS + LPS + MXC groups. For APP, Aβ, COX-2, DP2, and DP1 proteins, the relative optical density was normalized to β-actin. Each result of the NS group is normalized to 1. Data are mean ± SEM, *n* = 4. (* *p* < 0.05, ** *p* < 0.01, *** *p* < 0.001 vs. NS + CSF group; # *p* < 0.05, ## *p* < 0.01 vs. LPS + CSF group; ^ *p* < 0.05, ^^ *p* < 0.01 vs. NS + LPS group; @ *p* < 0.05, @@ *p* < 0.01 vs. LPS + LPS group).

To investigate the inhibitory effect of the meloxicam on brain damage via the inhibition of neuroinflammation, the protein expressions of APP, Aβ, COX-2, and DP2 in the cortex and hippocampus were also determined by Western blot analysis (Figure 5). The protein expressions of Aβ, COX-2, and DP2 in the cortex were obviously increased in LPS + CSF, NS + LPS, and LPS + LPS groups compared with the NS + CSF group, while the expression of DP1 was obviously decreased (*p* < 0.05 and *p* < 0.01). There was no significant difference in the protein expression of APP between the NS + CSF group and the LPS + CSF group. The expressions of APP, Aβ, COX-2, and DP2 were significantly increased in the NS + LPS and LPS + LPS groups (*p* < 0.05 and *p* < 0.01) compared with the LPS + CSF group, while the expression of DP1 was significantly decreased (*p* < 0.05 and *p* < 0.01). Moreover, the LPS + LPS group (*p* < 0.05 and *p* < 0.01) demonstrated that the expressions of APP, Aβ, COX-2, and DP2 were markedly increased compared with the NS + LPS group, while the expression of DP1 was significantly decreased. Compared to the LPS + LPS group, the expressions of Aβ, COX-2, and DP2 in the LPS + LPS + MXC group were dramatically decreased (*p* < 0.05 and *p* < 0.01) (Figure 5b).

### 2.7. Primary Cultured and Identification of Neurons

**Figure 6:**
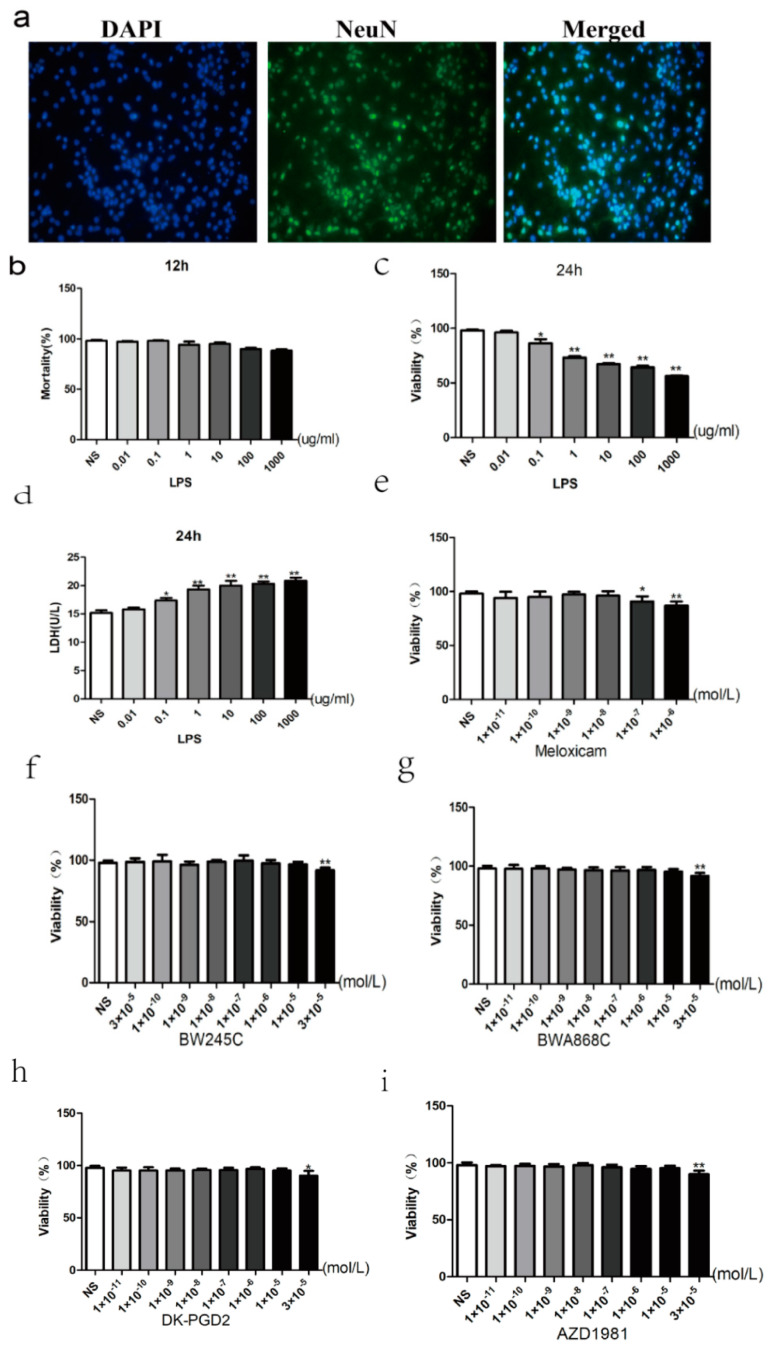
(**a**) Primary cultured and identification of neurons. (**b**,**c**) The effect of LPS on primary neurons. With the incremental concentration of LPS (12/24 h), the severe damage has been caused, and we also chose 1 ug/mL for the establishment of the damage model. (**d**) The change of LDH leakage rate intervened with LPS. (**e**) Meloxicam safety assessment. (**f**) BW245C safety assessment. (**g**) BWA868C safety assessment. (**h**) DK-PGD2 safety assessment. (**i**) AZD1981 safety assessment. Data is mean ± SEM, *n* = 6. (* *p* < 0.05, ** *p* < 0.01 vs. NS group)**.**

Observed under inverted microscope, the neuronal soma was plumped with short processes after cultured 1 day. Neurons appeared oval or vertebral after 3 days, and they had become stereoscopic. For seven days, the cultured neuronal bodies enlarge the nervous process’s eruption and extension into reticulation. The purity of neurons was identified by neuron-specific nuclear protein (NeuN) antibody and DAPI staining. Primary neurons grew well, and the purity was still up to 95% (Figure 6a).

### 2.8. Dose-Dependent Effect of LPS

To further understand the mechanisms underlying the regional difference in the sensitivity to LPS-induced neurotoxicity observed in vivo and its relationship to activity of neuron cultures, we used newborn rat brain tissues from the hippocampus and cortex. Viability of primary neurons were assessed by MTT assay. The viability of the control group was seen as 100%. In the extended data figure, cells incubated with a concentration gradient (0.01~1000 μg/mL) of LPS (12 or 24 h) revealed a dramatic reduction in the survival rate. The mortality rate of cells was almost 70%, while the dosage of LPS was between 10 and 1000 μg/mL. According to the previous studies and our results, 1 μg/mL LPS (24 h) was chosen for the damage model (Figure 6b,c). To further confirm the best concentration of LPS, we utilized the LDH assay and showed the same results (Figure 6d).

### 2.9. Effect of MXC on the Neuron Viability and the LDH Leakage Rate in LPS-Treated Primary Cultured Neurons of Offspring from Prenatal Inflammation Exposure

**Figure 7:**
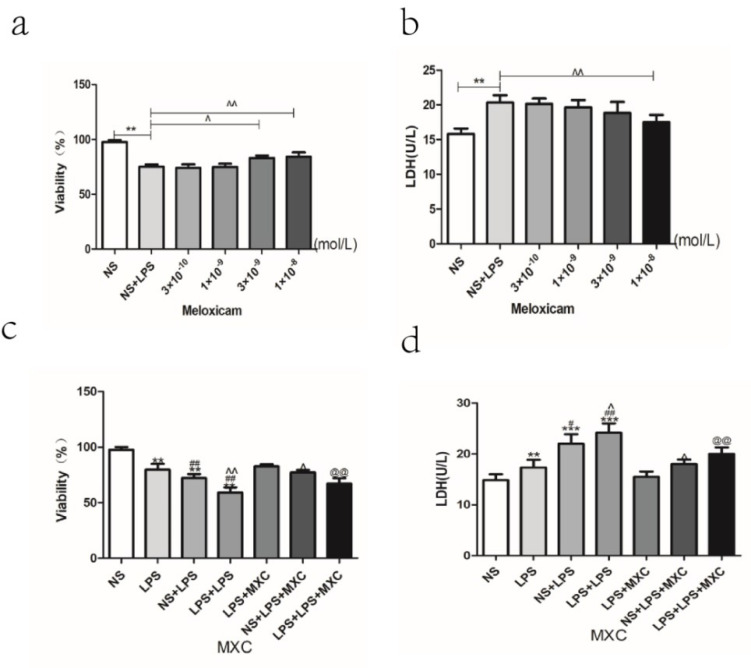
Effect of MXC on the neuron viability and the LDH leakage rate in LPS-treated primary cultured neurons of offspring from prenatal inflammation exposure: (**a**) Effect of MXC on the survival rate in LPS-treated neurons. (**b**) Effect of MXC on the LDH leakage rate in LPS-treated neurons. (**c**) Effect of MXC on the survival rate of LPS-treated primary neurons of offspring from prenatal inflammation exposure. (**d**) Effect of MXC on the LDH leakage rate in LPS-treated primary neurons of offspring from prenatal inflammation exposure. Data are mean ± SEM, *n* = 6. (** *p* < 0.01, *** *p* < 0.001 vs. NS group; # *p* < 0.05, ## *p* < 0.01 vs. LPS group; ^ *p* < 0.05, ^^ *p* < 0.01 vs. NS + LPS group; @@ *p* < 0.01 vs. LPS + LPS group).

MTT results showed that the concentration of 1 × 10^−6^ and 1 × 10^−7^ of meloxicam have toxic effects on normal primary cultured neurons, and that, for the subsequent experiment, we no longer use concentrations above 10^−7^ (Figure 6e). Immunofluorescence was used to detect the purity of the primary cultured rat neuron after being cultured for 7 days (Figure 6a). The dose-dependent effect of meloxicam on neuronal viability was detected by MTT. The results showed that the neuron viability decreased significantly in the LPS-treated group at the concentration of 1 × 10^−8^ and 1 × 10^−9^ mol/L, with neuron survival rates of 84.47% and 82.33%, respectively, compared with the control group. Therefore, we chose the best concentration of 1 × 10^−8^ as a follow-up experiment (Figure 7a). To further confirm the best concentration of meloxicam, we used the LDH assay and showed the same results (Figure 7b).

Compared with NS group, the viability of primary cultured neurons was significantly decreased in LPS, NS + LPS, and LPS + LPS groups (*p* < 0.01). Compared with LPS group, the viability of primary cultured neurons was significantly decreased in NS + LPS and LPS + LPS groups (*p* < 0.01). In particular, compared with NS + LPS group, the viability of primary cultured neurons was significantly decreased in the LPS + LPS group (*p* < 0.01). However, the neuron viability increased significantly in the meloxicam-treated group (Figure 7c). Compared with NS group, the rate of LDH leakage was significantly increased in LPS, NS + LPS, and LPS + LPS groups (*p* < 0.05 and *p* < 0.01). Compared with LPS group, the rate of LDH leakage was significantly increased in NS + LPS and LPS + LPS groups (*p* < 0.05 and *p* < 0.01). In particular, compared with the NS + LPS group, the rate of LDH leakage was significantly increased in the LPS + LPS group (*p* < 0.05). However, the rate of LDH leakage decreased significantly in the meloxicam-treated group (Figure 7d).

### 2.10. Effect of MXC on the Levels of TNF-α, IL-6, IL-1β, and PGD2 in LPS-Treated Primary Cultured Neurons of Offspring from Prenatal Inflammation Exposure

**Figure 8:**
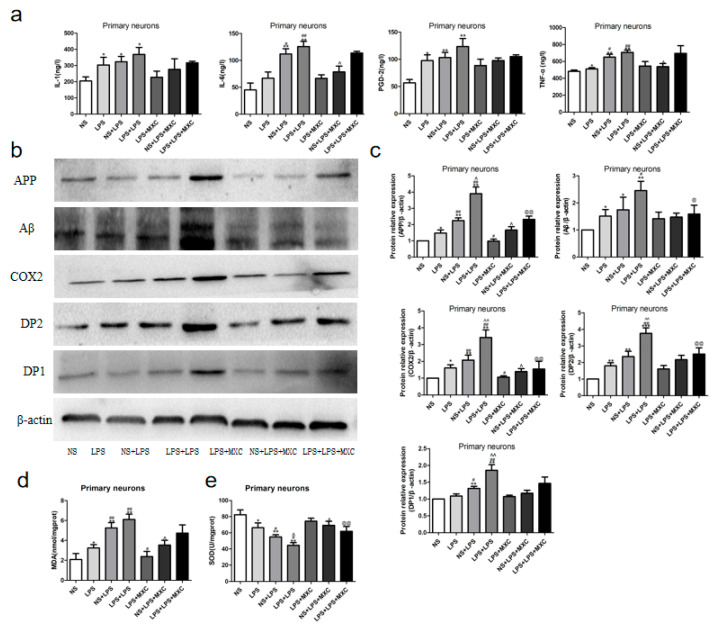
Effect of MXC on inflammatory response protein expressions and oxidative stress in LPS-treated primary neurons of offspring from prenatal inflammation exposure: (**a**) The levels of IL-1β, IL-6, PGD-2, and TNF-α in the primary cultured neurons (*n* = 6). (**b**,**c**) Western blots analysis of the relative protein contents in the primary cultured neurons. (**b**) The expression of APP, Aβ, COX-2, DP2, and DP1 was detected by Western blotting using specific antibodies in the primary neurons of rats; protein contents were plotted for NS + CSF, LPS + CSF, NS + LPS, LPS + LPS, LPS + CSF. MXC, NS + LPS + MXC, LPS + LPS + MXC. (**c**) The relative optical density was normalized to β-actin; Each result of the NS group is normalized to 1. (**d**,**e**) Effect of MXC on the levels of MDA and the activity of SOD in LPS-treated primary neurons of offspring after inflammation during pregnancy. (**d**) and the levels of MDA. (**e**) The activity of SOD. Data are mean ± SEM, *n* = 4. (* *p* < 0.05, ** *p* < 0.01, *** *p* < 0.001 vs. NS group, # *p* < 0.05, ## *p* < 0.01 vs. LPS group, ^ *p* < 0.05, ^^ *p* < 0.01 vs. NS + LPS group, @ *p* < 0.05, @@ *p* < 0.01 vs. LPS + LPS group)**.**

To further determine whether the COX-2/PGD-2/DPs pathway could participate in LPS-induced neuroinflammation, levels of IL-1β, IL-6, PGD-2, and TNF-α in the primary cultured neurons were considered by ELISA (Figure 8a). Compared with NS group, the levels of IL-1β, PGD-2, and TNF-α in the primary cultured neurons were significantly increased in LPS, NS + LPS, and LPS + LPS groups (*p* < 0.05 and *p* < 0.01), and no difference in the levels of IL-6 was observed between the NS group and the LPS group. Additionally, compared with the LPS group, the levels of TNF-α and IL-6 were significantly increased in the LPS + LPS group (*p* < 0.05); no difference in the levels of IL-1β and PGD-2 were observed. There was no significant difference in the levels of PGD-2, TNF-α, IL-1β, and IL-6 between the LPS group and the LPS + MXC group. Compared to the NS + LPS group, the levels of TNF-α and IL-6 were decreased significantly in the NS + LPS + MXC group (*p* < 0.05), whereas there was no significant difference in the levels of PGD-2 and IL-1β. There was no significant difference in the levels of PGD-2, TNF-α, IL-1β, and IL-6 between the LPS + LPS group and the LPS + LPS + MXC group (*p* < 0.05 and *p* < 0.01).

### 2.11. Effect of MXC on the Protein Expressions of COX-2, DP2, DP1, Aβ, and APP in LPS-Treated Primary Cultured Neurons of Offspring from Prenatal Inflammation Exposure

Based on the results of the protein expression analysis of animals, the protein of DP1 of LPS-treated primary neurons after inflammation during pregnancy was not similar in its trend. The expressions of APP, Aβ, COX-2, DP2, and DP1 in the LPS, NS + LPS, and LPS + LPS groups (*p* < 0.05 and *p* < 0.01), compared with the NS group, increased significantly (Figure 8b,c). The expressions of APP, Aβ, COX-2, DP2, and DP1 in NS + LPS and LPS + LPS groups (*p* < 0.05 and *p* < 0.01) were obviously increased compared with the LPS group. Moreover, the LPS + LPS group demonstrated that the expression of APP, Aβ, COX-2, DP2, and DP1 in the primary cultured neurons were significantly increased compared with the NS + LPS group (*p* < 0.05 and *p* < 0.01). Compared with the LPS group, the expressions of APP and COX-2 in the LPS + MXC group (*p* < 0.05) were dramatically decreased, whereas there was no significant difference in the protein expression of Aβ, DP2, and DP1. Compared with the NS + LPS group, the expressions of APP and COX-2 in the NS + LPS + MXC group (*p* < 0.05) were significantly decreased, whereas there was no significant difference in the protein expression of Aβ, DP2, and DP1. The expression of APP, Aβ, COX-2, and DP2 is lower after the administration of meloxicam than the LPS + LPS group (*p* < 0.05 and *p* < 0.01), whereas there was no significant difference in the protein expression of DP1 between the LPS + LPS group and the LPS + LPS + MXC group.

### 2.12. Effect of MXC on the Levels of MDA and the Activity of SOD in LPS-Treated Primary Cultured Neurons of Offspring from Prenatal Inflammation Exposure

As shown in Figure 8d, compared with NS group, the level of MDA in the primary cultured neurons was significantly increased in LPS, NS + LPS, and LPS + LPS groups (*p* < 0.05 and *p* < 0.01). Compared with LPS group, the level of MDA was obviously increased in NS + LPS and LPS + LPS groups (*p* < 0.05 and *p* < 0.01). Meanwhile, the level of MDA was significantly increased in the LPS + LPS group compared with the NS + LPS group (*p* < 0.05). Compared with LPS group, the level of MDA was decreased significantly in the LPS + MXC group (*p* < 0.05). Compared with NS + LPS group, the level of MDA was decreased significantly in the NS + LPS + MXC group (*p* < 0.05). There was no significant difference in the level of MDA between the LPS + LPS group and the LPS + LPS + MXC group.

As shown in Figure 8e, compared with the NS group, the activity of SOD in the primary neurons was significantly decreased in LPS, NS + LPS, and LPS + LPS groups (*p* < 0.05 and *p* < 0.01). The activity of SOD was significantly decreased in NS + LPS and LPS + LPS groups (*p* < 0.05 and *p* < 0.01) compared with the LPS group. Compared with the NS + LPS group, the activity of SOD in the primary neurons was significantly decreased in the LPS + LPS group (*p* < 0.05). There was no significant difference in the activity of SOD between the LPS group and the LPS + MXC group. Compared with the NS + LPS group, the activity of SOD was increased significantly in the NS + LPS + MXC group (*p* < 0.05). Compared with LPS + LPS group, the activity of SOD was increased significantly in the LPS + LPS + MXC group (*p* < 0.01).

### 2.13. Effect of DP Agonist or Antagonist on the Neuron Viability and the LDH Leakage in LPS-Treated Primary Cultured Neurons of Offspring from Prenatal Inflammation Exposure

**Figure 9:**
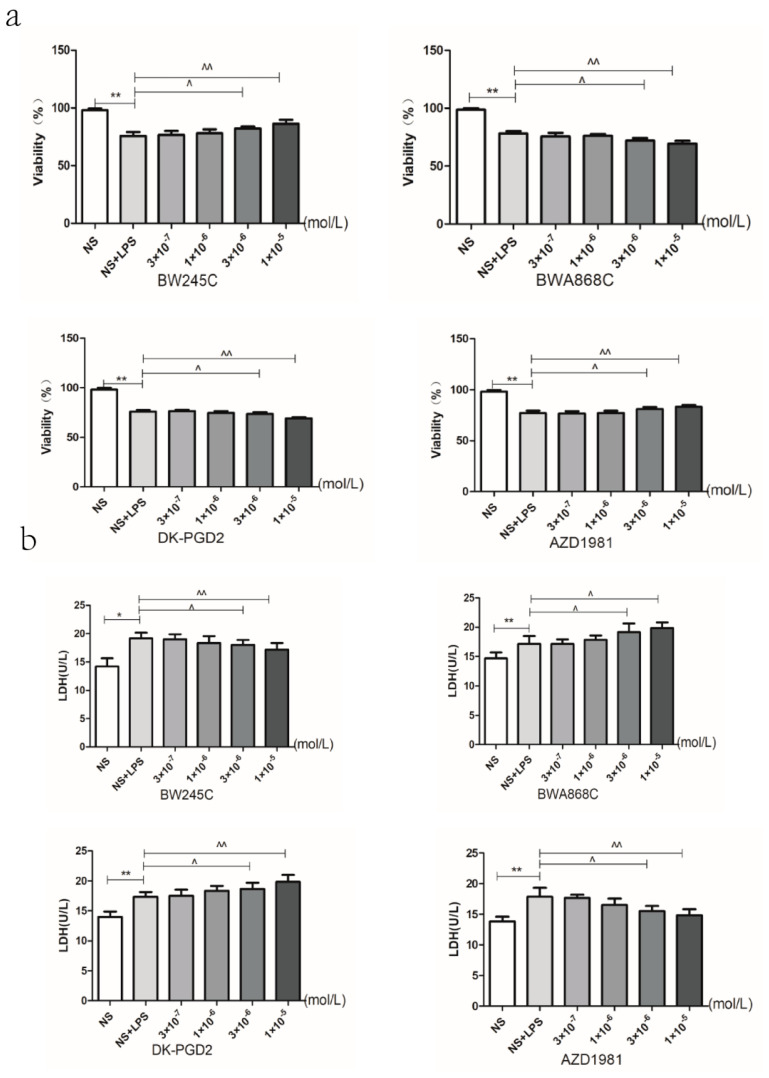

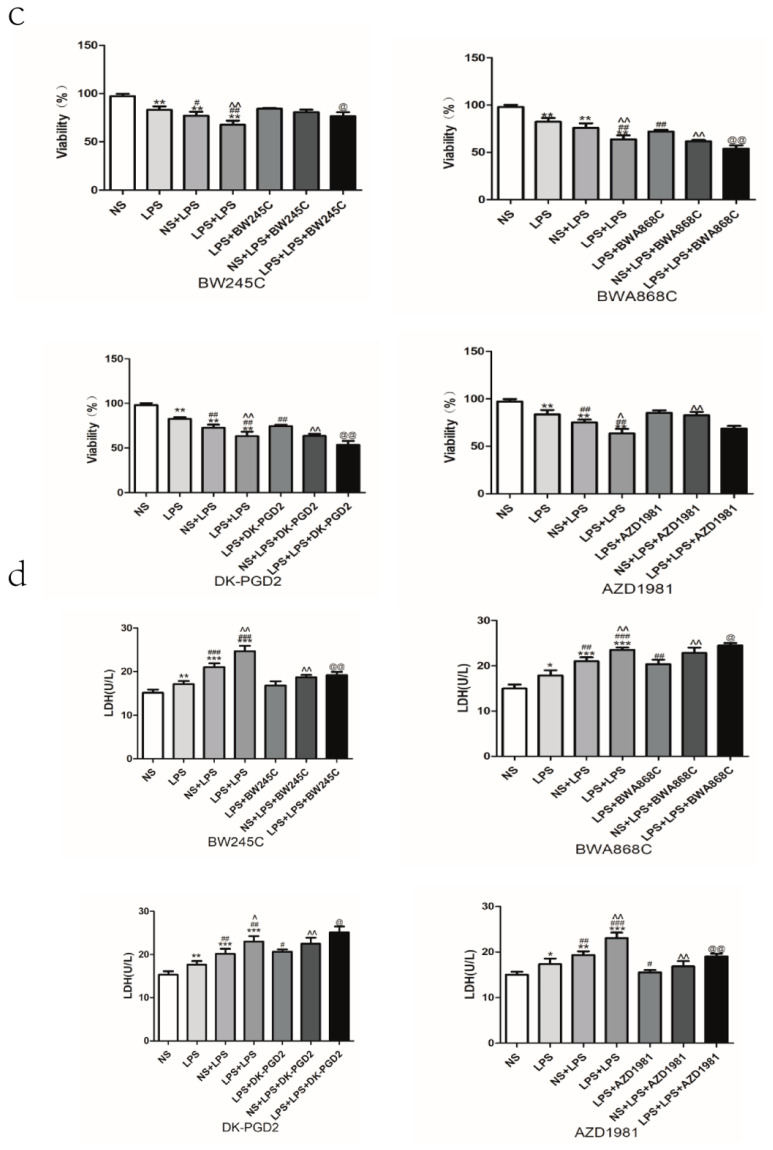
Effect of DP agonist or antagonist on the neuron viability and the LDH leakage in LPS-treated primary neurons of offspring from prenatal inflammation exposure: (**a**) Effect of DP agonists and antagonists on the changes of neuronal viability caused by LPS detected by the method of MTT. (**b**) The change of the LDH leakage rate intervened with the DP agonists and antagonists. (**c**) Change of neuronal viability intervened with the DP agonists and antagonists in each group. (**d**) The change of LDH leakage rate intervened with the DP agonists and antagonists in each group. Data are mean ± SEM, *n* = 6. (* *p* < 0.05, ** *p* < 0.01, *** *p* < 0.001 vs. NS group, # *p* < 0.05, ## *p* < 0.01, ### *p* < 0.001 vs. LPS group, ^ *p* < 0.05, ^^ *p* < 0.01 vs. NS + LPS group, @ *p* < 0.05, @@ *p* < 0.01 vs. LPS + LPS group).

MTT results showed that the concentration of 3 × 10^−5^ M of DP agonists and antagonists have toxic effects on normal primary neurons. Subsequent experiment is no longer in this concentration (Figure 6f–i). Regarding the concentrations of the agonists and antagonists of DP1 and DP2, for the observation of neuronal viability (Figure 9a) and the LDH leakage rate (Figure 9b), four concentrations (10^−5^, 3 × 10^−6^, 10^−6^, 3 × 10^−7^ M) of each agonists and antagonists of DP were used. The results showed that DP1 agonists (BW245C), DP2 antagonist (AZD1981), DP1 antagonist (BWA868C), and DP2 agonists (DK-PGD2) showed no significant cytotoxicity to primary cultured neurons at a concentration less than 10^−5^ M. Therefore, the concentration of 10^−5^ M for each agonist and antagonist of DP was chosen for the follow-up experiments.

Compared with NS group, the neuron viability was clearly decreased in the LPS, NS + LPS, and LPS + LPS groups (*p* < 0.05 and *p* < 0.01). The neuron viability was decreased significantly in the NS + LPS and LPS + LPS groups (*p* < 0.05 and *p* < 0.01) compared with the LPS group. Moreover, the neuron viability of the LPS + LPS group (*p* < 0.05) was decreased significantly compared with the NS + LPS group. The neuron viability increased significantly after the administration of DP1 agonists (BW245C) and the DP2 antagonist (AZD1981) (*p* < 0.05 and *p* < 0.01), whereas the DP1 antagonist (BWA868C) and the DP2 agonists (DK-PGD2) significantly decreased neuron viability (*p* < 0.05 and *p* < 0.01) (Figure 9c).

Compared with NS group, the rate of LDH leakage was clearly decreased in the LPS, NS + LPS, and LPS + LPS groups (*p* < 0.05 and *p* < 0.01). Significant differences in the rate of LDH leakage were witnessed between the LPS group and the LPS + LPS group (*p* < 0.01). In particular, the LDH leakage rate of the LPS + LPS group was increased significantly compared with the NS + LPS group (*p* < 0.05). The LDH leakage rate decreased markedly after the administration of DP1 agonists (BW245C) and the DP2 antagonist (AZD1981) (*p* < 0.05 and *p* < 0.01), whereas the DP1 antagonist (BWA868C) and DP2 agonists (DK-PGD2) significantly increased the LDH leakage rate (*p* < 0.05 and *p* < 0.01) (Figure 9d).

### 2.14. Effect of COX2 or DP Intervention on the Apoptosis in LPS-Treated Primary Cultured Neurons of Offspring from Prenatal Inflammation Exposure

**Figure 10:**
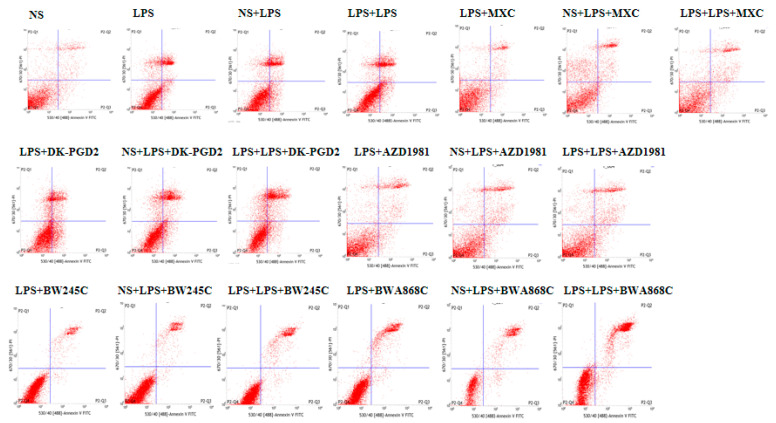
Determination of apoptosis cells with flow cytometry.

Primary neurons were treated with LPS for 24 h. LPS administration markedly elevated the level of apoptosis. Significant increases in the level of apoptosis in primary neurons were observed in the LPS, NS + LPS, and LPS + LPS groups compared with the NS group. Moreover, the LPS + LPS group demonstrated that the level of apoptosis in primary neurons was increased compared with the NS + LPS group in the primary neurons. The number of apoptotic cells decreased efficiently after the administration of MXC, DP1 agonists (BW245C), and DP2 antagonist (AZD1981), whereas the DP1 antagonist (BWA868C) and DP2 agonists (DK-PGD2) efficiently increased the number of apoptotic cells (Figure 10).

## 3. Discussion

It has been proposed that multiple infectious agents can cause inflammation, which may be a critical link between maternal infection and higher risk of neurodevelopmental disturbances [32]. However, little is known about whether prenatal maternal inflammation is involved in neuroinflammation and cognitive impairment in rat offspring. In this paper, we demonstrated that maternal LPS exposure during pregnancy caused memory impairment and neuronal necrosis. Moreover, prenatal LPS exposure caused the cultured neuronal death in vitro. Similar with our results, previous studies have shown that prenatal inflammation contributes to behavioral abnormalities associated with neurodevelopmental disorders in both primate and rodent offspring [15,33]. There are also studies supporting the concept that prenatal PolyI:C exposure results in long-lasting effects on hippocampus neurogenesis [34]. In addition, our findings revealed that maternal immune challenge leads to regulated cytokines expression in the cortex, such as upregulating PGD2, TNF-α, and oxidative stress index expression. It is closely related to maternally generated cytokines during the acute phase of prenatal LPS-induced acute inflammation, and challenge can enter the fetal circulation and increase cytokine levels in the fetal brain [35]. These increased cytokines can disrupt neuronal survival, differentiation, and apoptosis, interfere with the expression of transmitters and neurotrophins, and cause excitotoxicity in the developing brain [36]. Accordingly, acute cytokine exposure due to prenatal immune activation may act as a “vulnerability” factor for later-life memory impairment.

Related clinical studies have shown that Gram-negative bacterial LPS colocalized with Aβ1-40/42 in amyloid plaques and with Aβ1-40/42 around blood vessels in AD brains [9]. The involvement of inflammatory mechanisms in the pathogenesis of AD indicates that the combination of proinflammatory cytokine spectrum and diffuse amyloid deposition may initiate the process of self-reproduction, leading to the progression of the plaque and thus leading to increased risk of developing AD [37]. To investigate the long-term effects of prenatal immune challenge on neurobehavioral function following LPS-induced brain damage, a battery of neurobehavioral tasks were performed in rat offspring beginning at PD67. Here, our offspring rats with inflammation during pregnancy were injected with LPS lateral ventricle to mimic the pathophysiological processes of AD-like brain damage. Our finding revealed that “two-hit” rats (LPS + LPS) showed reduced numbers in exploratory activity and increased escape latency compared with “single-hit” rats (NS + LPS). Taken together, these results suggested that prenatal inflammation exposure exacerbated LPS-induced exploration as well as spatial learning impairment. HE staining revealed greater hippocampal neuronal damage in the “two-hit” rats compared with “single-hit” rats. The hippocampus plays a fundamental role in learning, memory, and emotional functioning [38]. Consequently, it is reasonable to predict that enhanced neuronal damage could be associated with exacerbated behavioral deficits after LPS-induced brain damage in the “two-hit” animals. In addition, our study shows that prenatal maternal inflammation exacerbated LPS-induced brain inflammatory response and significantly increased the protein expressions of Aβ and APP in the rat hippocampus and cortex. The same result occurred in vitro. APP is the source of extracellular amyloid-β plaques, which are believed to cause damage to neurons, especially to neuronal synapses [39]. Expressions of APP and Aβ has been shown to be increased in neuroinflammation and to be involved in cognitive impairment [40]. Thus, a prenatal immune challenge is sufficient to trigger a series of neuropathologic events that lead to a slow but gradual increase in amyloidogenic APP processing and cognitive impairments, potentially representing a state of increased vulnerability of the brain to AD.

The pathogeneses of cognitive impairment are complicated, such as excitotoxicity hypothesis, oxidative stress hypothesis, brain inflammation hypothesis, and Ca2 + overload hypothesis. However, neuroinflammation and oxidative stress seem to play prominent roles in enhancing the vulnerability of the AD-like brain damage [41]. Cakala et al. suggested that the early activation of COX-2 may be associated with Aβ oligomer accumulation in the brain, and that the COX-2 inhibitor protects the brain against Aβ-induced memory disturbances [42]. In an MPTP PD model in mice, COX-2 expression was considered to be associated with neuronal damage [43], thereafter leading to experiments that assessed the protective effects of COX-2 inhibitors against neurodegeneration [44]. After focal cerebral ischemia in rats, the protein expression of COX-2 was elevated, and COX-2 inhibitors significantly reduced infarct size, and protection was observed after 6 h of delayed administration [24]. The fact that COX-2 can be induced by inflammatory stimuli indicates that COX-2 may be regarded as the target for anti-inflammation [45]. Therefore, COX2 inhibitor (meloxicam) was used to investigate the relationship among COX-2, Aβ, and the susceptibility to brain damage in offspring rats of prenatal inflammation in our present study. As a result, the COX-2 inhibitor (meloxicam) significantly inhibited memory impairment, neuronal necrosis, oxidative stress, and inflammatory response, and significantly downregulated the expression of APP, Aβ, COX2, whereas it significantly increased exploring behavior. These results suggest that meloxicam exerts a significantly protective effect on the susceptibility to brain damage in the offspring of pregnant rats after inflammation. All in all, these results indicate that the increased susceptibility might be the important reason underlying brain tissue damage caused by an imbalance of COX-2-PGD2-DPs.

Prostaglandin D2, the most abundant prostanoid of the mammalian brain, has been shown to have roles in a variety of functions, from the regulation of sleep and temperature to inflammatory disorders [27]. There are two receptor subtypes for PGD-2, namely, DP1 receptor and DP2 receptor. Andreasson’s group showed that the DP1 agonist BW245C increases the level of cAMP and protects hippocampal slice cultures against N-methyl-D-aspartic acid (NMDA)-induced excitotoxicity [46] and the activation of the DP2 receptor, which is negatively coupled to cAMP via a pertussis toxin-sensitive mechanism [47]. It was speculated that DP1 plays a protective role in the brain damage model in excitability poisoning and ischemia, while DP2 accelerates the brain damage process. Interestingly, previous reports have suggested that DP1 knockout alleviated Th2-mediated respiratory inflammation in ovalbumin (OVA)-induced DP1^−/−^ mice asthma model, and suggested that PGD2 plays an inflammatory role in allergic inflammation through the DP1-mediated signal transduction pathway [48]. Aihua Zhang et al. found that DP2 antagonist BAY-u3405 does not play a protective role in PGD2, and inhibits the TGF-beta1-induced epithelial-to-mesenchymal transition [49]. Here, our data found that the content of PGD-2 and the protein expression of DP2 were significantly increased, while DP1 was significantly decreased in vivo. The protein expression of COX-2, DP2, and DP1 were significantly increased in vitro. However, the reason for the inconsistent expression of DP1 during in vivo and in vitro experiments is currently unclear, which requires further clarification.

Afterwards, we aimed to validate the effects of DP1 and DP2 in LPS-treated primary cultured neurons of offspring after prenatal inflammation exposure. To this end, we intervened with the PGD-2/DPs pathway of the DP receptor agonists and antagonists to clarify the importance of this pathway in LPS-treated primary neurons of offspring from prenatal inflammation exposure. In our study, compared with LPS + LPS treatment, after DP1 agonist (BW245C) or DP2 antagonist (CAY10471) intervention, neuron survival rate increased significantly and LDH leakage rate and cell apoptosis decreased significantly. However, after the administration of the DP1 antagonist (BWA868C) or DP2 agonist (DK-PGD2), neuron survival rate decreased significantly, and LDH leakage rate and cell apoptosis increased significantly. To our knowledge, DP1 stimulation can stimulate adenylate cyclase with a subsequent increase in intracellular cAMP, which increases the protein kinase A (PKA) level [50]. In contrast, relevant studies indicate that DP2 can activate intracellular transcription factors, regulate the expression of Th2 cytokines, and induce chemotactic changes to produce immune responses [51,52]. These results together show that DP2 may mediate the neurotoxicity of LPS-treated primary cultured neurons of offspring after prenatal inflammation exposure, while DP1 may mediate a protective mechanism. Thus, finding the mechanism of how DPs’ intervention leads to an improvement in functional outcome, possibly by increasing cAMP/PKA-regulated gene expression and decreasing the inflammatory response, is necessary to develop a new candidate target into a potential therapy. However, considering the complexity of this downstream pathway, the specific regulation mechanism of DPs in the central nervous system is worth further study.

## 4. Materials and Methods

### 4.1. Animals

Sprague–Dawley (SD) rats were purchased from Laboratory Animal Center of Chongqing Medical University and housed in the barrier housing facility, in accordance with national standard “Laboratory Animal-Requirements of Environment and Housing Facilities” and the National Institutes of Health guidelines. The care of laboratory animal and the animal experimental operation conformed “Chongqing Administration Rule of Laboratory Animal”. The experimental procedures were approved by the animal laboratory administrative center and the institutional ethics committee of Chongqing Medical University (License number: SYXK YU 2012-0001). Animals were given ad libitum access to food and water. Females were transferred to new cages (2/cage) and male bedding added two days prior to male presentation [53]. Male rats were housed 1:2 females for one or two days until the vaginal plug appeared, which was considered embryonic day 0 (E0), and each female was moved into a new cage with nesting material.

### 4.2. Establishment of Animal Models

In this experiment, paired rats (220–250 g) were checked daily for vaginal plugs, indicating pregnancy. Pregnant females (*n* = 30) were housed singly and were injected intraperitoneally (i.p.) with lipopolysaccharide (LPS) (Escherichia coli, serotype 055: B5; Sigma–Aldrich, St Louis, MO, USA) diluted in normal saline (NS) at the dose of 300 µg/kg on gestational day (GD) 11, 14, and 18, respectively [54,55]. The control group (*n* = 9) consisted of pregnant rats injected with normal saline on the same days.

Pups from the experimental and control groups were fed until postnatal day (PD) 60. All rats were anesthetized by 50 mg/kg of 1% pentobarbital sodium (i.p.). Then, brain damage susceptibility in response to LPS in male offspring was tested on PD 60. Next, 60 µg LPS in 5 µL sterile or vehicle intra-cerebroventricular injections were performed once at a rate of 1 µL/min with a 10 µL Hamilton Syringe operated by a peristaltic pump. To perform the injection into the lateral ventricle, the following coordinates were used: 1 mm posterior from bregma, 1.8 mm laterals from the sagittal suture, and 3.5 mm below the dura12, [56].

### 4.3. Design and Treatment Groups

On PD 60, male offspring of LPS or NS-treated mothers were injected bilateral lateral ventricle with LPS (12 µg/µL, 5 µL) or cerebrospinal fluid (CSF) (5 µL; Beijing Bioolab Technology Co. Ltd. Cat#: GL0304). Control rats received comparable volume of CSF. Seven experimental groups were studied, including normal control (NS + CSF group), prenatal infection (LPS + CSF group), postnatal infection (NS + LPS group), prenatal and postnatal infection (LPS + LPS group), LPS + CSF + MXC group, NS + LPS + MXC group, and LPS + LPS + MXC group, with *n* = 10 in each group. MXC stands for the drug of meloxicam (a COX2 inhibitor; Beijing Solarbio; M9840). During the experimental period, only 1 rat died in the LPS + LPS + MXC group. No obvious abnormalities in surrounding tissues and organs were found in the autopsy of the dead rat. Therefore, there were at least 9 rats remaining in each group when the administration was completed.

### 4.4. Morris Water Maze Test

To evaluate spatial learning and memory impairment, we injected LPS in offspring after exposure to inflammation during pregnancy. Rats were treated with meloxicam 24 h after LPS injection. Seven days after the continuous administration of meloxicam, we performed the Morris water maze test to observe the cognitive function of the rats. Morris water maze (Beijing Zhongshi Di Chuang Technology Development Co., Ltd.; Beijing, China) was used to measure spatial learning and memory function of rats in each group [57]. Briefly, rats received four trials per day for four consecutive days. A different entry site was used for each daily session. During each trial, the rats were included in the water where a hidden platform was submerged under the water. If rats failed to reach the platform within 90 s, they were gently guided and allowed to remain on the platform for 10 s. On the fifth day, following the last day of training, rats were introduced into the pool from the entry site, where the last training was performed in order to assess retention of the platform location. During this probe trial, the platform was removed from the maze. The latency to find the hidden platform, the trajectory of motion, and the amount of time crossing the platform were recorded, with a maximum of 90 s.

### 4.5. Open-Field Test (OFT)

The open field test (OFT) was used to assess spontaneous activity levels and the curiosity of rats in the original environment (Figure 2). It is an accepted behavioral tool in measuring the exploratory behavior and spontaneous activity in rodents. Repetitive grooming rats displayed an abnormal spontaneous repetitive behavior pattern and high levels of repetitive self-grooming. Open-field test was conducted on rats 9 days after bilateral lateral ventricle injection of LPS or CSF, using a test device of 100 cm square black enclosure with 30 cm tall walls set on a non-reflective black base. This open-field apparatus was located in a small, quiet room [58,59]. Rats were habituated in the testing room inside their home cages and left undisturbed for 1 min prior to testing. The inner surface of the test apparatus was painted with black. The floor of the apparatus (100 cm × 100 cm × 40 cm) was divided into 25 identical squares (20 cm × 20 cm) with white stripes.

In a dark (visibility was 5 m) and quiet room, a single rat was placed at the center of the arena and allowed to explore for 5 min. We recorded the number of squares crossed by the rats (with all four paws crossing the line, each line cross was scored as 1 point), the rearing behaviors (each rearing was scored as 1 point), the grooming behaviors (each groom point; the urine was also scored as 1 point), and the latency of locomotion. After each of these tests, the excrement was cleaned, and the arena was treated with 75% alcohol. The score was calculated by the sum.

### 4.6. Rat Autonomic Activity Test

The rats were placed in a YLS-1B Multifunctional rat Autonomic Activity Recorder (Beijing Zhongxi Yuanda Technology Co., Ltd., SJ-YLS-1B, Beijing, China), and, after 3 min of acclimatization, the number of activities in rats within 5 min was observed and recorded. Autonomous activity experiments were performed in a quiet laboratory at 25 °C at 19:00 pm [60].

### 4.7. Histopathological Observation

After the Morris water maze test, 3 rats from each group were perfused with heparinized saline (30 mL) to remove blood from the vasculature, and then with 4% paraformaldehyde in phosphate buffered saline (50 mL). The whole brain was then removed and stored in the same fixative. After paraffin embedding, 5µm sections were obtained and stained with hematoxylin-eosin (HE). Morphologic changes in hippocampus and cortical neurons were examined using light microscopy. High-power fields were sampled from the hippocampus CA1 subfield. Cells with a distinct nucleus and nucleolus were regarded as intact neurons [32]. The number of necrotic neuronal injured cells was counted and expressed as a percentage for each hippocampal or cortical region.

### 4.8. Primary Neuron Culture

Primary neuron was prepared on PD 0 from SD rats by previous methods [61], with minor modifications. Briefly, the neonatal rat was sterilized by 75% ethyl alcohol and then sacrificed; the brain was collected on ice plate. After the removal of meningeal and vessels, the cortex and hippocampus were separated by ophthalmic forceps and put into the plate with dissecting fluid. The cortex and hippocampus were cut into 1 mm^3^ and were then washed twice by dissecting fluid. The tissues were digested at 37 °C for 20 min in pancreatic enzyme solution, which was stopped by DMEM/F12 medium with 10% fetal bovine serum (FBS) (Gibco, Carlsbad, CA, USA). We then added 2 ml culture solution, beat upon for 10~15 times, made the cell suspension, standing the cell suspension for 1 min, absorbed supernatant into another tube, added 2 mL cultivation liquid, repeated percussion, and then combined the supernatant. The supernatant was filtered through a 200-mesh sieve. Next, 25 µL filtered liquids was added with 475 µL cultivation liquid, diluted for 20 times, then used for counting. Cells were diluted to 1 × 107/mL by cultivation liquid and inoculate into the plate. After 4 h, the culture medium was replaced with neurobasal medium supplemented with 2% B-27 (Gibco, Carlsbad, CA, USA), and half of the medium was changed every 3 days. After being cultivated for 48 h, nerve cells were treated with 4 mg/mL cytarabine (ARA-C), which could inhibit the excessive growth of glial cells, cultivated for 7 days, and then can be used for the study.

### 4.9. Characterization of Neuron by Immunofluorescence

Cells were fixed with 4% paraformaldehyde for 30 min; non-specific binding was blocked by incubating cells in a 5% BSA and 0.1% Triton X-100 solution for 1 h at room temperature. The primary neurons were incubated with rabbit NeuN polyclonal antibody (1:100) in the blocking solution overnight at 4 °C. After three washes with PBS, the primary neurons were incubated with the corresponding FITC-conjugated goat anti-rabbit IgG (1:200) for 2 h at room temperature, and the nuclei were stained with DAPI. Slides were viewed under a fluorescence microscope (Nikon, Inc., Tokyo, Japan) [62].

### 4.10. Establishment of the Model of Primary Cultured Neuron Damage Induced by LPS

On the 7th day, primary neurons were treated with serial concentrations of LPS (0.01 µg/mL, 0.1 µg/mL, 1 µg/mL, 10 µg/mL, 100 µg/mL, or 1000 µg/mL) for 12 or 24 h. Cell viability was determined by MTT (Sigma-Aldrich(Shanghai) Trading Co, Ltd., Shanghai, China) assay, and the optimum concentration of LPS for damage model was figured out by the MTT assay results [40]. Cell viability (%) = (OD of cells with different treatment)/(OD of cells with solution medium) × 100.

### 4.11. Detection of Neuron Viability

The primary cultured primary neuronal viability was determined by MTT assay. Neurons were plated in 96-well culture plates at a density of 1 × 107 neurons/mL. After treatment, the culture supernatant was discarded, and 100 µL of 5 mg/mL MTT was added to each well. The media was carefully removed, and the color was developed after incubation with 150 µL DMSO for 4 h. Finally, absorbance (OD) was read at 570 nm by a microplate reader (BioTek, Winooski, VT, USA) [63].

In order to determine the relationship between LPS damage to neuron and COX-2 pathway, MXC was used, and cells were divided into seven groups: NS, LPS, NS + LPS, LPS + LPS, LPS + MXC, NS + LPS + MXC, and LPS + LPS + MXC groups. Among them, NS group was normal control; LPS group was the primary neurons of offspring from prenatal inflammation exposure; the NS + LPS group was the LPS-treated primary neurons of offspring from normal pregnant rats; LPS + LPS group was given LPS double stimulation for pregnancy inflammation offspring; *n* = 6 in each group.

To observe the effect of the PGD2-DP pathway on the LPS-treated primary cultured neurons of offspring from prenatal inflammation exposure, the neurons were divided into NS, LPS, NS + LPS, LPS + LPS, LPS + drug, NS + LPS + drug, and LPS + LPS + drug groups. Drugs including DP1 agonist (BW245C), DP1 antagonist (BWA868C), DP2 agonist (DK-PGD2), and DP2 antagonist (CAY10471), which were added up into the intervention group, respectively.

### 4.12. LDH Leakage Rate Assay

Cultured rat primary neurons in the 96-well culture plate were cultured until D7 for drug treatment. Cells were quantitated by the measurement of extracellular lactate dehydrogenase (LDH, Beyotime, Shanghai, China) activity using an LDH-Cytotoxicity test (WAKO), as recommended by the manufacturer. Briefly, primary neurons were seeded onto a 96-well plate, pretreated with or without CM. Released LDH activity was measured by adding reaction reagent comprising nitro blue tetrazolium (NBT) diaphorase and nicotinamide adenine dinucleotide (NAD) to produce a colored diformazan. After 45 min, 100 µL of 1 M HCl was added to each sample to stop the reaction. Then, the absorbance, which reflects cell damage, was detected with a colorimeter (Viento, Dainippon, Tokyo, Japan) at 560 nm [64]. The lactate dehydrogenase (LDH) leakage rate was measured.

### 4.13. Analysis of Neuronal Apoptosis by Flow Cytometry

Apoptosis of neurons was identified using an Annexin-V and PI apoptosis kit (BU-ap0102, Life Technologies, Carlsbad, CA, USA). After washing twice with PBS, cells were resuspended in binding buffer and incubated with Annexin-V and PI for 15 min. At least 5000 cells were counted per sample. Apoptotic cells appeared as Annexin-V positive and PI negative [65].

### 4.14. Oxidative Stress Level Measurement

The level of Malondialdehyde (MDA) and the activity of superoxide dismutase (SOD), as two signs of lipid peroxidation, were measured using the test kit (Nanjing Jiancheng Bioengineering Institute, A001-1, Nanjing, China) by a modified procedure illustrated by Al-Amin, M. M., et al. (2016) [61]. The protein content was measured using the BCA protein assay kit (Beyotime, Shanghai, China).

### 4.15. Biochemical Assay

The levels of TNF-α, IL-1β, IL-6, and PGD-2 in brain tissue extracts (*n* = 4), cell supernatants, and culture medium were measured with ELISA kit (Jiangsu Mei Biao Biological Technology Co., Ltd., Yancheng, China) [66].

### 4.16. Western Blotting Test

Next, 50 mg of rat cortex or hippocampus (*n* = 4) was added to 0.5 mL of tissue lysate solution for protein extraction; primary culture neurons from each group were collected and lysed by RIPA Lysis Buffer on ice. Then, centrifugation at 12,000× *g* for 10 min at 4 °C, and the supernatant was used for the detection of protein concentrations with a BCA protein assay kit (Beyotime, Shanghai, China). Several 10µL samples of protein were separated by sodium dodecyl sulphate polyacrylamide gel electrophoresis (SDS-PAGE) and transferred to PVDF membranes (Millipore, Burlington, MA, USA). The membranes were blocked with 5% BSA for 1 h at room temperature and then probed with specific primary antibodies, including anti-Aβ (1:1000; Abcam, Cambridge, UK), COX-2 (1:1000; Abcam, Cambridge, UK), APP (1:1000; BOSTER, Shanghai, China), DP1 (1:1000; Abcam, Cambridge, UK), DP2 (1:500; Santa, San Antonio, TX, USA), and β-actin (1:4000; Proteintech, Chicago, IL, USA) overnight at 4 °C. The membranes were washed three times in TBST and incubated with HRP-conjugated secondary antibodies at room temperature for 1 h. Following four washes in TBST, protein signals were visualized by ECL (Bio-Rad, Hercules, CA, USA).

### 4.17. Statistical Analysis

All data analyses were made using GraphPad Prism 5.01(San Diego, CA, USA, www.graphpad.com, accessed on April 2019). Data are shown as mean ± SEM. One-way ANONVA and the Tukey test were used to compare the measurements among three or more groups, and a t-test was used to compare the measurements between two groups. Statistical significance was set at *p* < 0.05.

## 5. Conclusions

Our results reveal that inflammatory stimuli during pregnancy can cause dysplasia and neuronal inflammation in offspring. Maternal LPS exposure during pregnancy leads to increased susceptibility of brain damage to LPS in offspring. The COX-2/PGD-2/DP pathway plays a major role in brain changes induced by prenatal exposure to inflammation and the increased susceptibility of brain damage, which could be a target for disease-modifying approaches in brain damage.

## Data Availability

The data that support the present results are available from the corresponding author upon reasonable request.
